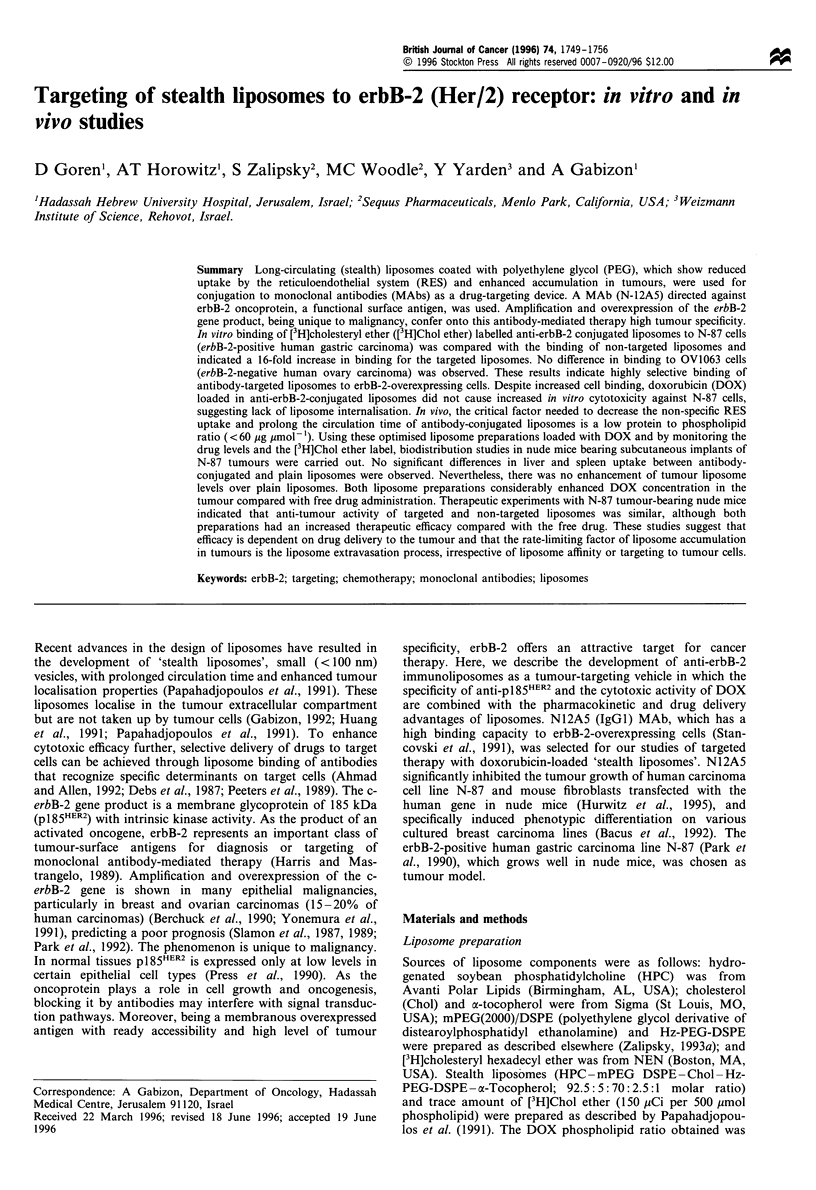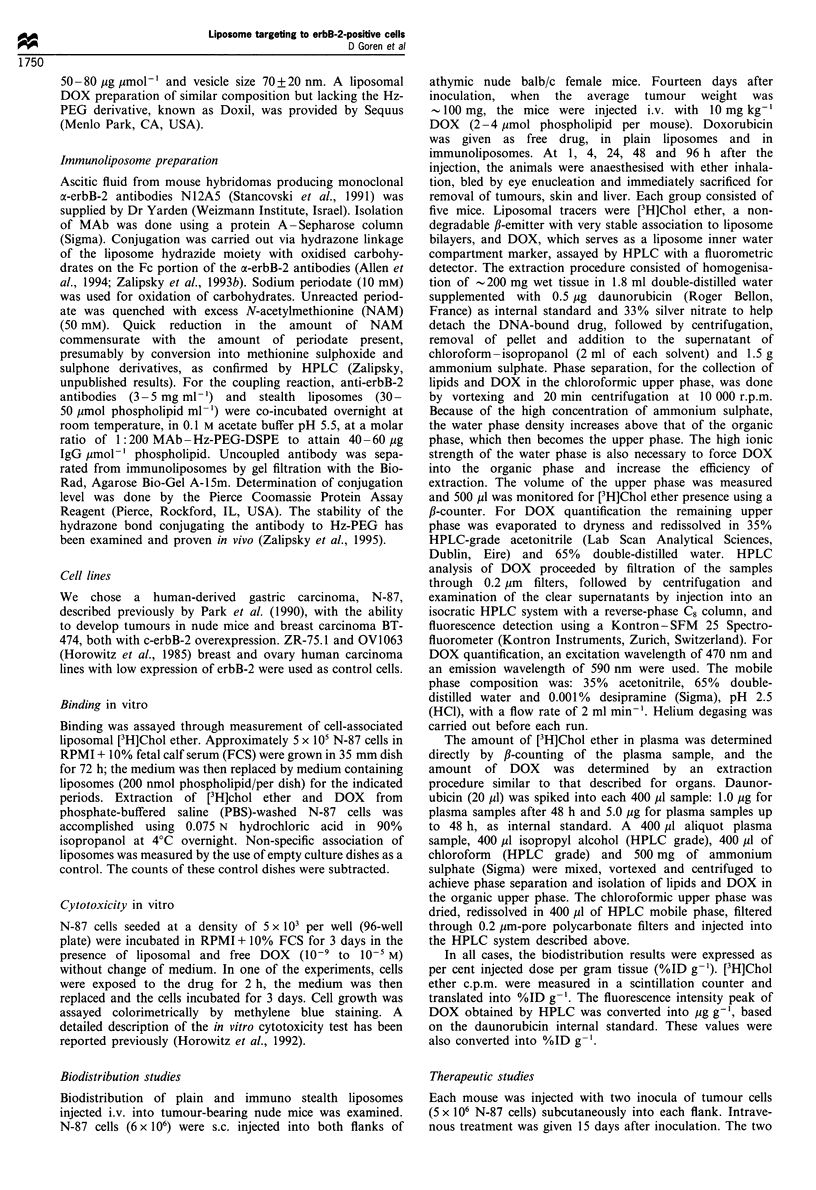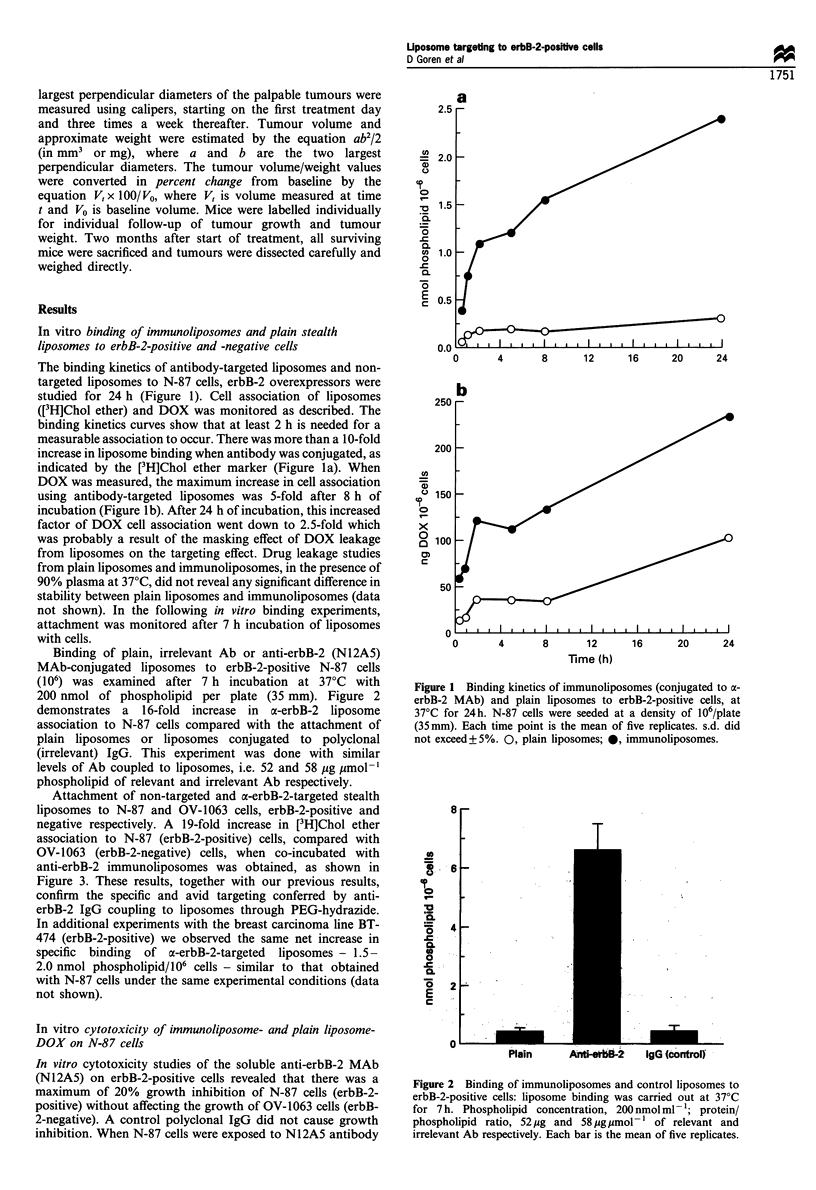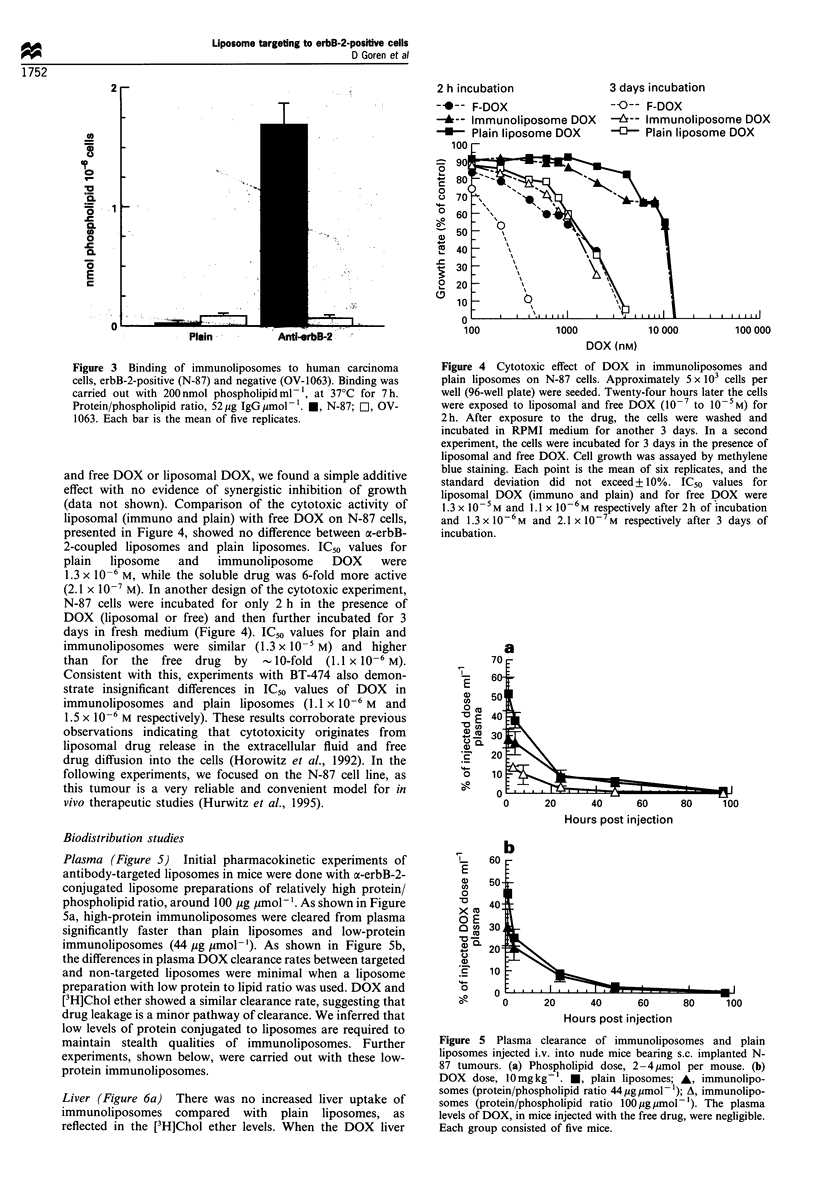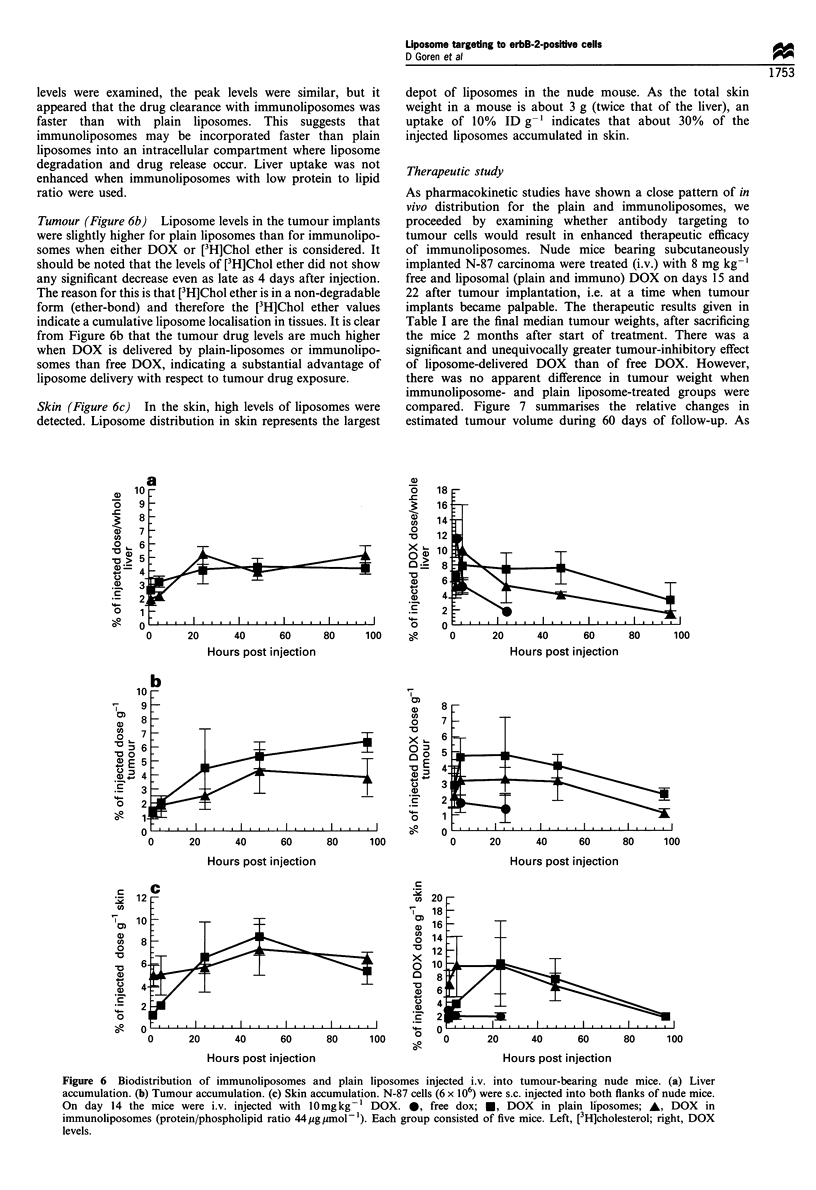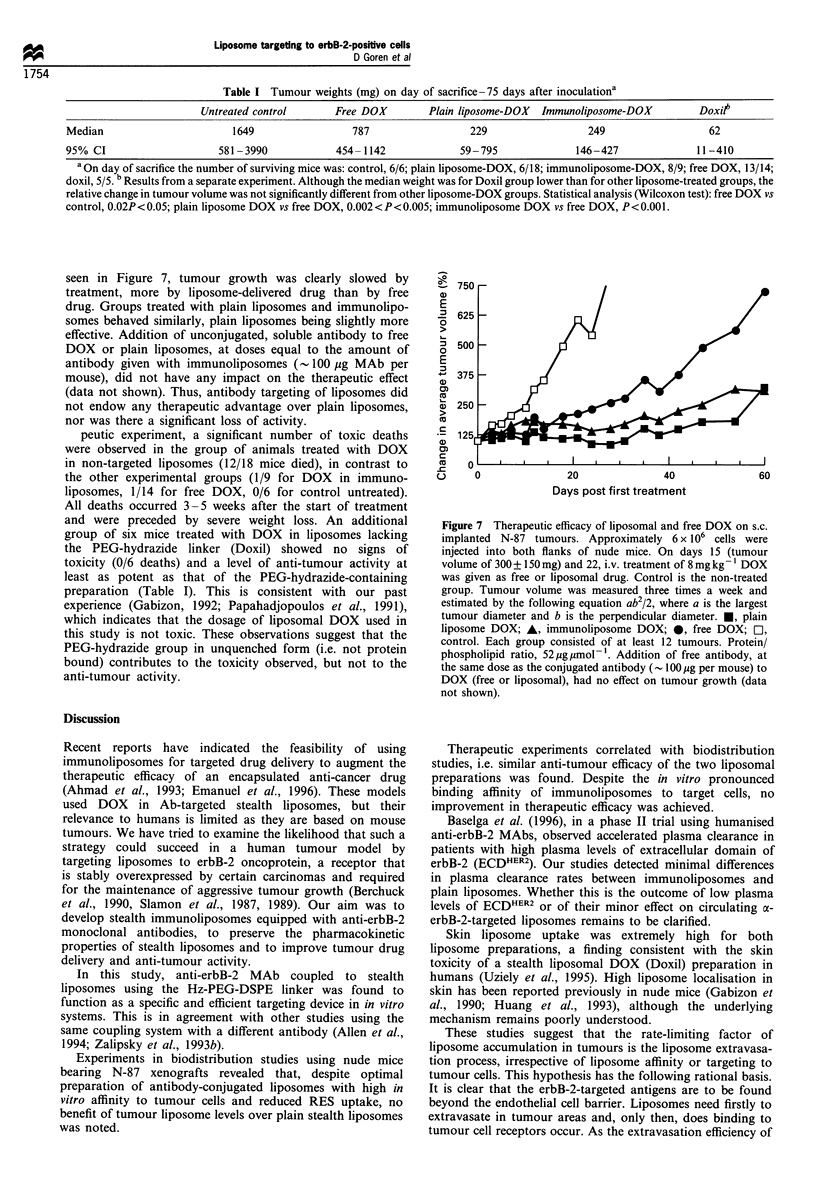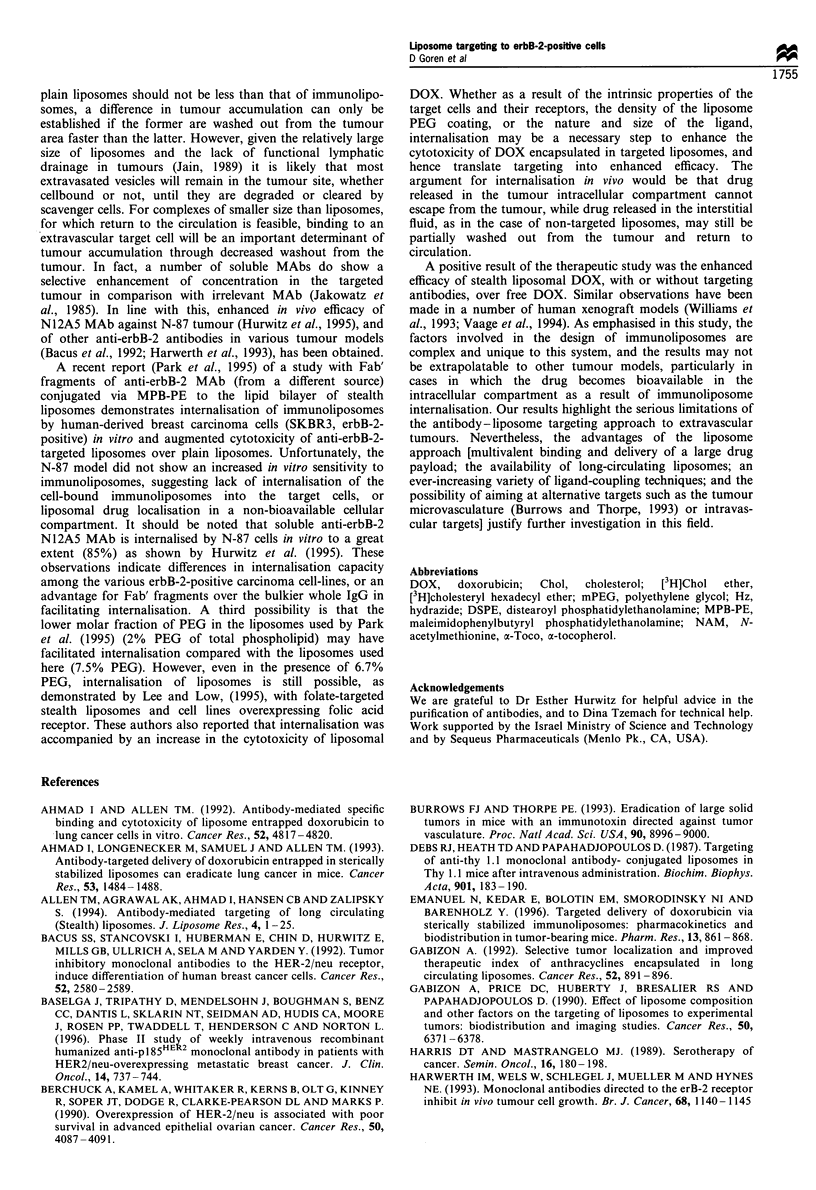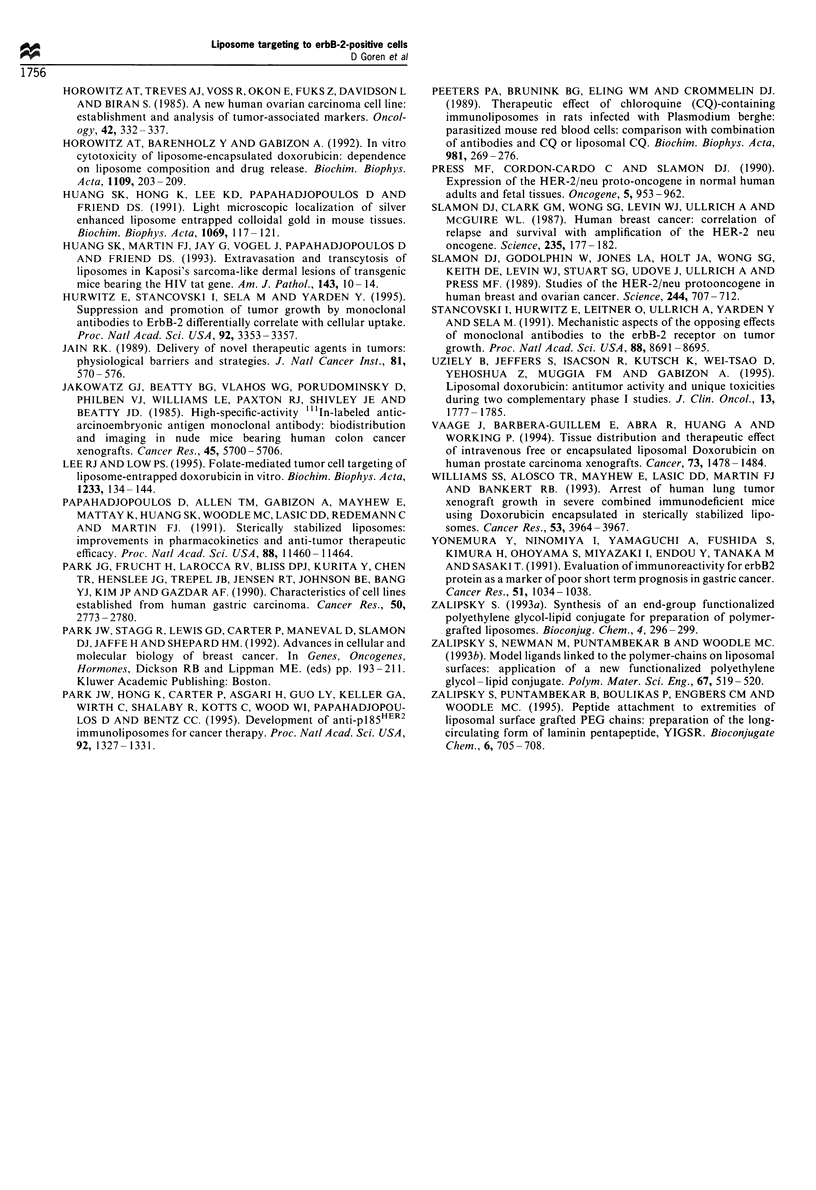# Targeting of stealth liposomes to erbB-2 (Her/2) receptor: in vitro and in vivo studies.

**DOI:** 10.1038/bjc.1996.625

**Published:** 1996-12

**Authors:** D. Goren, A. T. Horowitz, S. Zalipsky, M. C. Woodle, Y. Yarden, A. Gabizon

**Affiliations:** Hadassah Hebrew University Hospital, Jerusalem, Israel.

## Abstract

Long-circulating (stealth) liposomes coated with polyethylene glycol (PEG), which show reduced uptake by the reticuloendothelial system (RES) and enhanced accumulation in tumours, were used for conjugation to monoclonal antibodies (MAbs) as a drug-targeting device. A MAb (N-12A5) directed against erbB-2 oncoprotein, a functional surface antigen, was used. Amplification and overexpression of the erbB-2 gene product, being unique to malignancy, confer onto this antibody-mediated therapy high tumour specificity. In vitro binding of [3H]cholesteryl ether ([3H]Chol ether) labelled anti-erbB-2 conjugated liposomes to N-87 cells (erbB-2-positive human gastric carcinoma) was compared with the binding of non-targeted liposomes and indicated a 16-fold increase in binding for the targeted liposomes. No difference in binding to OV1063 cells (erbB-2-negative human ovary carcinoma) was observed. These results indicate highly selective binding of antibody-targeted liposomes to erbB-2-overexpressing cells. Despite increased cell binding, doxorubicin (DOX) loaded in anti-erbB-2-conjugated liposomes did not cause increased in vitro cytotoxicity against N-87 cells, suggesting lack of liposome internalisation. In vivo, the critical factor needed to decrease the non-specific RES uptake and prolong the circulation time of antibody-conjugated liposomes is a low protein to phospholipid ratio ( < 60 micrograms mumol-1). Using these optimised liposome preparations loaded with DOX and by monitoring the drug levels and the [3H]Chol ether label, biodistribution studies in nude mice bearing subcutaneous implants of N-87 tumours were carried out. No significant differences in liver and spleen uptake between antibody-conjugated and plain liposomes were observed. Nevertheless, there was no enhancement of tumour liposome levels over plain liposomes. Both liposome preparations considerably enhanced DOX concentration in the tumour compared with free drug administration. Therapeutic experiments with N-87 tumour-bearing nude mice indicated that anti-tumour activity of targeted and non-targeted liposomes was similar, although both preparations had an increased therapeutic efficacy compared with the free drug. These studies suggest that efficacy is dependent on drug delivery to the tumour and that the rate-limiting factor of liposome accumulation in tumours is the liposome extravasation process, irrespective of liposome affinity or targeting to tumour cells.


British Journal of Cancer (1996) 74, 1749-1756
© 1996 Stockton Press All rights reserved 0007-0920/96 $12.00

### Targeting of stealth liposomes to erbB-2 (Her/2) receptor: *in vitro* and *in vivo* studies

D Goren[1], AT Horowitz[1], S Zalipsky[2], MC Woodle[2], Y Yarden[3] and A Gabizon[1]

[1]*Hadassah Hebrew University Hospital, Jerusalem, Israel;* [2]*Sequus Pharmaceuticals, Menlo Park, California, USA;* [3]*Weizmann Institute of Science, Rehovot, Israel.*

**Summary** Long-circulating (stealth) liposomes coated with polyethylene glycol (PEG), which show reduced uptake by the reticuloendothelial system (RES) and enhanced accumulation in tumours, were used for conjugation to monoclonal antibodies (MAbs) as a drug-targeting device. A MAb (N-12A5) directed against erbB-2 oncoprotein, a functional surface antigen, was used. Amplification and overexpression of the *erb*B-2 gene product, being unique to malignancy, confer onto this antibody-mediated therapy high tumour specificity. *In vitro* binding of [$^3$H]cholesteryl ether ([$^3$H]Chol ether) labelled anti-erbB-2 conjugated liposomes to N-87 cells (*erb*B-2-positive human gastric carcinoma) was compared with the binding of non-targeted liposomes and indicated a 16-fold increase in binding for the targeted liposomes. No difference in binding to OV1063 cells (*erb*B-2-negative human ovary carcinoma) was observed. These results indicate highly selective binding of antibody-targeted liposomes to erbB-2-overexpressing cells. Despite increased cell binding, doxorubicin (DOX) loaded in anti-erbB-2-conjugated liposomes did not cause increased *in vitro* cytotoxicity against N-87 cells, suggesting lack of liposome internalisation. *In vivo*, the critical factor needed to decrease the non-specific RES uptake and prolong the circulation time of antibody-conjugated liposomes is a low protein to phospholipid ratio ($<60\ \mu g\ \mu mol^{-1}$). Using these optimised liposome preparations loaded with DOX and by monitoring the drug levels and the [$^3$H]Chol ether label, biodistribution studies in nude mice bearing subcutaneous implants of N-87 tumours were carried out. No significant differences in liver and spleen uptake between antibody-conjugated and plain liposomes were observed. Nevertheless, there was no enhancement of tumour liposome levels over plain liposomes. Both liposome preparations considerably enhanced DOX concentration in the tumour compared with free drug administration. Therapeutic experiments with N-87 tumour-bearing nude mice indicated that anti-tumour activity of targeted and non-targeted liposomes was similar, although both preparations had an increased therapeutic efficacy compared with the free drug. These studies suggest that efficacy is dependent on drug delivery to the tumour and that the rate-limiting factor of liposome accumulation in tumours is the liposome extravasation process, irrespective of liposome affinity or targeting to tumour cells.

**Keywords:** erbB-2; targeting; chemotherapy; monoclonal antibodies; liposomes

Recent advances in the design of liposomes have resulted in the development of 'stealth liposomes', small (<100 nm) vesicles, with prolonged circulation time and enhanced tumour localisation properties (Papahadjopoulos *et al.*, 1991). These liposomes localise in the tumour extracellular compartment but are not taken up by tumour cells (Gabizon, 1992; Huang *et al.*, 1991; Papahadjopoulos *et al.*, 1991). To enhance cytotoxic efficacy further, selective delivery of drugs to target cells can be achieved through liposome binding of antibodies that recognize specific determinants on target cells (Ahmad and Allen, 1992; Debs *et al.*, 1987; Peeters *et al.*, 1989). The c-*erb*B-2 gene product is a membrane glycoprotein of 185 kDa (p185$^{HER2}$) with intrinsic kinase activity. As the product of an activated oncogene, erbB-2 represents an important class of tumour-surface antigens for diagnosis or targeting of monoclonal antibody-mediated therapy (Harris and Mastrangelo, 1989). Amplification and overexpression of the c-*erb*B-2 gene is shown in many epithelial malignancies, particularly in breast and ovarian carcinomas (15–20% of human carcinomas) (Berchuck *et al.*, 1990; Yonemura *et al.*, 1991), predicting a poor prognosis (Slamon *et al.*, 1987, 1989; Park *et al.*, 1992). The phenomenon is unique to malignancy. In normal tissues p185$^{HER2}$ is expressed only at low levels in certain epithelial cell types (Press *et al.*, 1990). As the oncoprotein plays a role in cell growth and oncogenesis, blocking it by antibodies may interfere with signal transduction pathways. Moreover, being a membranous overexpressed antigen with ready accessibility and high level of tumour specificity, erbB-2 offers an attractive target for cancer therapy. Here, we describe the development of anti-erbB-2 immunoliposomes as a tumour-targeting vehicle in which the specificity of anti-p185$^{HER2}$ and the cytotoxic activity of DOX are combined with the pharmacokinetic and drug delivery advantages of liposomes. N12A5 (IgG1) MAb, which has a high binding capacity to erbB-2-overexpressing cells (Stancovski *et al.*, 1991), was selected for our studies of targeted therapy with doxorubicin-loaded 'stealth liposomes'. N12A5 significantly inhibited the tumour growth of human carcinoma cell line N-87 and mouse fibroblasts transfected with the human gene in nude mice (Hurwitz *et al.*, 1995), and specifically induced phenotypic differentiation on various cultured breast carcinoma lines (Bacus *et al.*, 1992). The erbB-2-positive human gastric carcinoma line N-87 (Park *et al.*, 1990), which grows well in nude mice, was chosen as tumour model.

### Materials and methods

### *Liposome preparation*

Sources of liposome components were as follows: hydrogenated soybean phosphatidylcholine (HPC) was from Avanti Polar Lipids (Birmingham, AL, USA); cholesterol (Chol) and α-tocopherol were from Sigma (St Louis, MO, USA); mPEG(2000)/DSPE (polyethylene glycol derivative of distearoylphosphatidyl ethanolamine) and Hz-PEG-DSPE were prepared as described elsewhere (Zalipsky, 1993*a*); and [$^3$H]cholesteryl hexadecyl ether was from NEN (Boston, MA, USA). Stealth liposomes (HPC–mPEG DSPE–Chol–Hz-PEG-DSPE–α-Tocopherol; 92.5:5:70:2.5:1 molar ratio) and trace amount of [$^3$H]Chol ether (150 μCi per 500 μmol phospholipid) were prepared as described by Papahadjopoulos *et al.* (1991). The DOX phospholipid ratio obtained was

Correspondence: A Gabizon, Department of Oncology, Hadassah Medical Centre, Jerusalem 91120, Israel
Received 22 March 1996; revised 18 June 1996; accepted 19 June 1996

50–80 $\mu$g $\mu$mol$^{-1}$ and vesicle size 70±20 nm. A liposomal DOX preparation of similar composition but lacking the Hz-PEG derivative, known as Doxil, was provided by Sequus (Menlo Park, CA, USA).

*Immunoliposome preparation*

Ascitic fluid from mouse hybridomas producing monoclonal $\alpha$-erbB-2 antibodies N12A5 (Stancovski *et al.*, 1991) was supplied by Dr Yarden (Weizmann Institute, Israel). Isolation of MAb was done using a protein A–Sepharose column (Sigma). Conjugation was carried out via hydrazone linkage of the liposome hydrazide moiety with oxidised carbohydrates on the Fc portion of the $\alpha$-erbB-2 antibodies (Allen *et al.*, 1994; Zalipsky *et al.*, 1993*b*). Sodium periodate (10 mM) was used for oxidation of carbohydrates. Unreacted periodate was quenched with excess *N*-acetylmethionine (NAM) (50 mM). Quick reduction in the amount of NAM commensurate with the amount of periodate present, presumably by conversion into methionine sulphoxide and sulphone derivatives, as confirmed by HPLC (Zalipsky, unpublished results). For the coupling reaction, anti-erbB-2 antibodies (3–5 mg ml$^{-1}$) and stealth liposomes (30–50 $\mu$mol phospholipid ml$^{-1}$) were co-incubated overnight at room temperature, in 0.1 M acetate buffer pH 5.5, at a molar ratio of 1:200 MAb–Hz-PEG-DSPE to attain 40–60 $\mu$g IgG $\mu$mol$^{-1}$ phospholipid. Uncoupled antibody was separated from immunoliposomes by gel filtration with the Bio-Rad, Agarose Bio-Gel A-15m. Determination of conjugation level was done by the Pierce Coomassie Protein Assay Reagent (Pierce, Rockford, IL, USA). The stability of the hydrazone bond conjugating the antibody to Hz-PEG has been examined and proven *in vivo* (Zalipsky *et al.*, 1995).

*Cell lines*

We chose a human-derived gastric carcinoma, N-87, described previously by Park *et al.* (1990), with the ability to develop tumours in nude mice and breast carcinoma BT-474, both with c-erbB-2 overexpression. ZR-75.1 and OV1063 (Horowitz *et al.*, 1985) breast and ovary human carcinoma lines with low expression of erbB-2 were used as control cells.

*Binding* in vitro

Binding was assayed through measurement of cell-associated liposomal [$^3$H]Chol ether. Approximately $5\times10^5$ N-87 cells in RPMI + 10% fetal calf serum (FCS) were grown in 35 mm dish for 72 h; the medium was then replaced by medium containing liposomes (200 nmol phospholipid/per dish) for the indicated periods. Extraction of [$^3$H]chol ether and DOX from phosphate-buffered saline (PBS)-washed N-87 cells was accomplished using 0.075 N hydrochloric acid in 90% isopropanol at 4°C overnight. Non-specific association of liposomes was measured by the use of empty culture dishes as a control. The counts of these control dishes were subtracted.

*Cytotoxicity* in vitro

N-87 cells seeded at a density of $5\times10^3$ per well (96-well plate) were incubated in RPMI + 10% FCS for 3 days in the presence of liposomal and free DOX ($10^{-9}$ to $10^{-5}$ M) without change of medium. In one of the experiments, cells were exposed to the drug for 2 h, the medium was then replaced and the cells incubated for 3 days. Cell growth was assayed colorimetrically by methylene blue staining. A detailed description of the *in vitro* cytotoxicity test has been reported previously (Horowitz *et al.*, 1992).

*Biodistribution studies*

Biodistribution of plain and immuno stealth liposomes injected i.v. into tumour-bearing nude mice was examined. N-87 cells ($6\times10^6$) were s.c. injected into both flanks of athymic nude balb/c female mice. Fourteen days after inoculation, when the average tumour weight was ~100 mg, the mice were injected i.v. with 10 mg kg$^{-1}$ DOX (2–4 $\mu$mol phospholipid per mouse). Doxorubicin was given as free drug, in plain liposomes and in immunoliposomes. At 1, 4, 24, 48 and 96 h after the injection, the animals were anaesthesised with ether inhalation, bled by eye enucleation and immediately sacrificed for removal of tumours, skin and liver. Each group consisted of five mice. Liposomal tracers were [$^3$H]Chol ether, a non-degradable $\beta$-emitter with very stable association to liposome bilayers, and DOX, which serves as a liposome inner water compartment marker, assayed by HPLC with a fluorometric detector. The extraction procedure consisted of homogenisation of ~200 mg wet tissue in 1.8 ml double-distilled water supplemented with 0.5 $\mu$g daunorubicin (Roger Bellon, France) as internal standard and 33% silver nitrate to help detach the DNA-bound drug, followed by centrifugation, removal of pellet and addition to the supernatant of chloroform–isopropanol (2 ml of each solvent) and 1.5 g ammonium sulphate. Phase separation, for the collection of lipids and DOX in the chloroformic upper phase, was done by vortexing and 20 min centrifugation at 10 000 r.p.m. Because of the high concentration of ammonium sulphate, the water phase density increases above that of the organic phase, which then becomes the upper phase. The high ionic strength of the water phase is also necessary to force DOX into the organic phase and increase the efficiency of extraction. The volume of the upper phase was measured and 500 $\mu$l was monitored for [$^3$H]Chol ether presence using a $\beta$-counter. For DOX quantification the remaining upper phase was evaporated to dryness and redissolved in 35% HPLC-grade acetonitrile (Lab Scan Analytical Sciences, Dublin, Eire) and 65% double-distilled water. HPLC analysis of DOX proceeded by filtration of the samples through 0.2 $\mu$m filters, followed by centrifugation and examination of the clear supernatants by injection into an isocratic HPLC system with a reverse-phase $C_8$ column, and fluorescence detection using a Kontron–SFM 25 Spectrofluorometer (Kontron Instruments, Zurich, Switzerland). For DOX quantification, an excitation wavelength of 470 nm and an emission wavelength of 590 nm were used. The mobile phase composition was: 35% acetonitrile, 65% double-distilled water and 0.001% desipramine (Sigma), pH 2.5 (HCl), with a flow rate of 2 ml min$^{-1}$. Helium degasing was carried out before each run.

The amount of [$^3$H]Chol ether in plasma was determined directly by $\beta$-counting of the plasma sample, and the amount of DOX was determined by an extraction procedure similar to that described for organs. Daunorubicin (20 $\mu$l) was spiked into each 400 $\mu$l sample: 1.0 $\mu$g for plasma samples after 48 h and 5.0 $\mu$g for plasma samples up to 48 h, as internal standard. A 400 $\mu$l aliquot plasma sample, 400 $\mu$l isopropyl alcohol (HPLC grade), 400 $\mu$l of chloroform (HPLC grade) and 500 mg of ammonium sulphate (Sigma) were mixed, vortexed and centrifuged to achieve phase separation and isolation of lipids and DOX in the organic upper phase. The chloroformic upper phase was dried, redissolved in 400 $\mu$l of HPLC mobile phase, filtered through 0.2 $\mu$m-pore polycarbonate filters and injected into the HPLC system described above.

In all cases, the biodistribution results were expressed as per cent injected dose per gram tissue (%ID g$^{-1}$). [$^3$H]Chol ether c.p.m. were measured in a scintillation counter and translated into %ID g$^{-1}$. The fluorescence intensity peak of DOX obtained by HPLC was converted into $\mu$g g$^{-1}$, based on the daunorubicin internal standard. These values were also converted into %ID g$^{-1}$.

*Therapeutic studies*

Each mouse was injected with two inocula of tumour cells ($5\times10^6$ N-87 cells) subcutaneously into each flank. Intravenous treatment was given 15 days after inoculation. The two largest perpendicular diameters of the palpable tumours were measured using calipers, starting on the first treatment day and three times a week thereafter. Tumour volume and approximate weight were estimated by the equation $ab^2/2$ (in $mm^3$ or mg), where $a$ and $b$ are the two largest perpendicular diameters. The tumour volume/weight values were converted in *percent change* from baseline by the equation $V_t \times 100/V_0$, where $V_t$ is volume measured at time $t$ and $V_0$ is baseline volume. Mice were labelled individually for individual follow-up of tumour growth and tumour weight. Two months after start of treatment, all surviving mice were sacrificed and tumours were dissected carefully and weighed directly.

### Results

### In vitro *binding of immunoliposomes and plain stealth liposomes to erbB-2-positive and -negative cells*

The binding kinetics of antibody-targeted liposomes and non-targeted liposomes to N-87 cells, erbB-2 overexpressors were studied for 24 h (Figure 1). Cell association of liposomes ([$^3$H]Chol ether) and DOX was monitored as described. The binding kinetics curves show that at least 2 h is needed for a measurable association to occur. There was more than a 10-fold increase in liposome binding when antibody was conjugated, as indicated by the [$^3$H]Chol ether marker (Figure 1a). When DOX was measured, the maximum increase in cell association using antibody-targeted liposomes was 5-fold after 8 h of incubation (Figure 1b). After 24 h of incubation, this increased factor of DOX cell association went down to 2.5-fold which was probably a result of the masking effect of DOX leakage from liposomes on the targeting effect. Drug leakage studies from plain liposomes and immunoliposomes, in the presence of 90% plasma at 37°C, did not reveal any significant difference in stability between plain liposomes and immunoliposomes (data not shown). In the following *in vitro* binding experiments, attachment was monitored after 7 h incubation of liposomes with cells.

Binding of plain, irrelevant Ab or anti-erbB-2 (N12A5) MAb-conjugated liposomes to erbB-2-positive N-87 cells ($10^6$) was examined after 7 h incubation at 37°C with 200 nmol of phospholipid per plate (35 mm). Figure 2 demonstrates a 16-fold increase in α-erbB-2 liposome association to N-87 cells compared with the attachment of plain liposomes or liposomes conjugated to polyclonal (irrelevant) IgG. This experiment was done with similar levels of Ab coupled to liposomes, i.e. 52 and 58 μg $\mu mol^{-1}$ phospholipid of relevant and irrelevant Ab respectively.

Attachment of non-targeted and α-erbB-2-targeted stealth liposomes to N-87 and OV-1063 cells, erbB-2-positive and negative respectively. A 19-fold increase in [$^3$H]Chol ether association to N-87 (erbB-2-positive) cells, compared with OV-1063 (erbB-2-negative) cells, when co-incubated with anti-erbB-2 immunoliposomes was obtained, as shown in Figure 3. These results, together with our previous results, confirm the specific and avid targeting conferred by anti-erbB-2 IgG coupling to liposomes through PEG-hydrazide. In additional experiments with the breast carcinoma line BT-474 (erbB-2-positive) we observed the same net increase in specific binding of α-erbB-2-targeted liposomes – 1.5–2.0 nmol phospholipid/$10^6$ cells – similar to that obtained with N-87 cells under the same experimental conditions (data not shown).

### In vitro *cytotoxicity of immunoliposome- and plain liposome-DOX on N-87 cells*

*In vitro* cytotoxicity studies of the soluble anti-erbB-2 MAb (N12A5) on erbB-2-positive cells revealed that there was a maximum of 20% growth inhibition of N-87 cells (erbB-2-positive) without affecting the growth of OV-1063 cells (erbB-2-negative). A control polyclonal IgG did not cause growth inhibition. When N-87 cells were exposed to N12A5 antibody

**Figure 1** Binding kinetics of immunoliposomes (conjugated to α-erbB-2 MAb) and plain liposomes to erbB-2-positive cells, at 37°C for 24h. N-87 cells were seeded at a density of $10^6$/plate (35mm). Each time point is the mean of five replicates. s.d. did not exceed ±5%. ○, plain liposomes; ●, immunoliposomes.

**Figure 2** Binding of immunoliposomes and control liposomes to erbB-2-positive cells: liposome binding was carried out at 37°C for 7h. Phospholipid concentration, 200 nmol $ml^{-1}$; protein/phospholipid ratio, 52 μg and 58 μg $\mu mol^{-1}$ of relevant and irrelevant Ab respectively. Each bar is the mean of five replicates.

**Figure 3** Binding of immunoliposomes to human carcinoma cells, erbB-2-positive (N-87) and negative (OV-1063). Binding was carried out with 200 nmol phospholipid ml$^{-1}$, at 37°C for 7 h. Protein/phospholipid ratio, 52 $\mu$g IgG $\mu$mol$^{-1}$. ■, N-87; □, OV-1063. Each bar is the mean of five replicates.

**Figure 4** Cytotoxic effect of DOX in immunoliposomes and plain liposomes on N-87 cells. Approximately $5 \times 10^3$ cells per well (96-well plate) were seeded. Twenty-four hours later the cells were exposed to liposomal and free DOX ($10^{-7}$ to $10^{-5}$ M) for 2 h. After exposure to the drug, the cells were washed and incubated in RPMI medium for another 3 days. In a second experiment, the cells were incubated for 3 days in the presence of liposomal and free DOX. Cell growth was assayed by methylene blue staining. Each point is the mean of six replicates, and the standard deviation did not exceed $\pm 10\%$. IC$_{50}$ values for liposomal DOX (immuno and plain) and for free DOX were $1.3 \times 10^{-5}$ M and $1.1 \times 10^{-6}$ M respectively after 2 h of incubation and $1.3 \times 10^{-6}$ M and $2.1 \times 10^{-7}$ M respectively after 3 days of incubation.

and free DOX or liposomal DOX, we found a simple additive effect with no evidence of synergistic inhibition of growth (data not shown). Comparison of the cytotoxic activity of liposomal (immuno and plain) with free DOX on N-87 cells, presented in Figure 4, showed no difference between $\alpha$-erbB-2-coupled liposomes and plain liposomes. IC$_{50}$ values for plain liposome and immunoliposome DOX were $1.3 \times 10^{-6}$ M, while the soluble drug was 6-fold more active ($2.1 \times 10^{-7}$ M). In another design of the cytotoxic experiment, N-87 cells were incubated for only 2 h in the presence of DOX (liposomal or free) and then further incubated for 3 days in fresh medium (Figure 4). IC$_{50}$ values for plain and immunoliposomes were similar ($1.3 \times 10^{-5}$ M) and higher than for the free drug by $\sim$10-fold ($1.1 \times 10^{-6}$ M). Consistent with this, experiments with BT-474 also demonstrate insignificant differences in IC$_{50}$ values of DOX in immunoliposomes and plain liposomes ($1.1 \times 10^{-6}$ M and $1.5 \times 10^{-6}$ M respectively). These results corroborate previous observations indicating that cytotoxicity originates from liposomal drug release in the extracellular fluid and free drug diffusion into the cells (Horowitz *et al.*, 1992). In the following experiments, we focused on the N-87 cell line, as this tumour is a very reliable and convenient model for *in vivo* therapeutic studies (Hurwitz *et al.*, 1995).

### *Biodistribution studies*

*Plasma (Figure 5)* Initial pharmacokinetic experiments of antibody-targeted liposomes in mice were done with $\alpha$-erbB-2-conjugated liposome preparations of relatively high protein/phospholipid ratio, around 100 $\mu$g $\mu$mol$^{-1}$. As shown in Figure 5a, high-protein immunoliposomes were cleared from plasma significantly faster than plain liposomes and low-protein immunoliposomes (44 $\mu$g $\mu$mol$^{-1}$). As shown in Figure 5b, the differences in plasma DOX clearance rates between targeted and non-targeted liposomes were minimal when a liposome preparation with low protein to lipid ratio was used. DOX and [$^3$H]Chol ether showed a similar clearance rate, suggesting that drug leakage is a minor pathway of clearance. We inferred that low levels of protein conjugated to liposomes are required to maintain stealth qualities of immunoliposomes. Further experiments, shown below, were carried out with these low-protein immunoliposomes.

*Liver (Figure 6a)* There was no increased liver uptake of immunoliposomes compared with plain liposomes, as reflected in the [$^3$H]Chol ether levels. When the DOX liver

**Figure 5** Plasma clearance of immunoliposomes and plain liposomes injected i.v. into nude mice bearing s.c. implanted N-87 tumours. (**a**) Phospholipid dose, 2–4 $\mu$mol per mouse. (**b**) DOX dose, 10 mg kg$^{-1}$. ■, plain liposomes; ▲, immunoliposomes (protein/phospholipid ratio 44 $\mu$g $\mu$mol$^{-1}$); △, immunoliposomes (protein/phospholipid ratio 100 $\mu$g $\mu$mol$^{-1}$). The plasma levels of DOX, in mice injected with the free drug, were negligible. Each group consisted of five mice.

levels were examined, the peak levels were similar, but it appeared that the drug clearance with immunoliposomes was faster than with plain liposomes. This suggests that immunoliposomes may be incorporated faster than plain liposomes into an intracellular compartment where liposome degradation and drug release occur. Liver uptake was not enhanced when immunoliposomes with low protein to lipid ratio were used.

*Tumour (Figure 6b)* Liposome levels in the tumour implants were slightly higher for plain liposomes than for immunoliposomes when either DOX or [$^3$H]Chol ether is considered. It should be noted that the levels of [$^3$H]Chol ether did not show any significant decrease even as late as 4 days after injection. The reason for this is that [$^3$H]Chol ether is in a non-degradable form (ether-bond) and therefore the [$^3$H]Chol ether values indicate a cumulative liposome localisation in tissues. It is clear from Figure 6b that the tumour drug levels are much higher when DOX is delivered by plain-liposomes or immunoliposomes than free DOX, indicating a substantial advantage of liposome delivery with respect to tumour drug exposure.

*Skin (Figure 6c)* In the skin, high levels of liposomes were detected. Liposome distribution in skin represents the largest depot of liposomes in the nude mouse. As the total skin weight in a mouse is about 3 g (twice that of the liver), an uptake of 10% ID g$^{-1}$ indicates that about 30% of the injected liposomes accumulated in skin.

### *Therapeutic study*

As pharmacokinetic studies have shown a close pattern of *in vivo* distribution for the plain and immunoliposomes, we proceeded by examining whether antibody targeting to tumour cells would result in enhanced therapeutic efficacy of immunoliposomes. Nude mice bearing subcutaneously implanted N-87 carcinoma were treated (i.v.) with 8 mg kg$^{-1}$ free and liposomal (plain and immuno) DOX on days 15 and 22 after tumour implantation, i.e. at a time when tumour implants became palpable. The therapeutic results given in Table I are the final median tumour weights, after sacrificing the mice 2 months after start of treatment. There was a significant and unequivocally greater tumour-inhibitory effect of liposome-delivered DOX than of free DOX. However, there was no apparent difference in tumour weight when immunoliposome- and plain liposome-treated groups were compared. Figure 7 summarises the relative changes in estimated tumour volume during 60 days of follow-up. As

**Figure 6** Biodistribution of immunoliposomes and plain liposomes injected i.v. into tumour-bearing nude mice. (**a**) Liver accumulation. (**b**) Tumour accumulation. (**c**) Skin accumulation. N-87 cells ($6 \times 10^6$) were s.c. injected into both flanks of nude mice. On day 14 the mice were i.v. injected with 10 mg kg$^{-1}$ DOX. ●, free dox; ■, DOX in plain liposomes; ▲, DOX in immunoliposomes (protein/phospholipid ratio 44 $\mu$g $\mu$mol$^{-1}$). Each group consisted of five mice. Left, [$^3$H]cholesterol; right, DOX levels.

**Table I** Tumour weights (mg) on day of sacrifice–75 days after inoculation[a]

| | *Untreated control* | *Free DOX* | *Plain liposome-DOX* | *Immunoliposome-DOX* | *Doxil*[b] |
|---|---|---|---|---|---|
| Median | 1649 | 787 | 229 | 249 | 62 |
| 95% CI | 581–3990 | 454–1142 | 59–795 | 146–427 | 11–410 |

[a] On day of sacrifice the number of surviving mice was: control, 6/6; plain liposome-DOX, 6/18; immunoliposome-DOX, 8/9; free DOX, 13/14; doxil, 5/5. [b] Results from a separate experiment. Although the median weight was for Doxil group lower than for other liposome-treated groups, the relative change in tumour volume was not significantly different from other liposome-DOX groups. Statistical analysis (Wilcoxon test): free DOX *vs* control, $0.02P<0.05$; plain liposome DOX *vs* free DOX, $0.002<P<0.005$; immunoliposome DOX *vs* free DOX, $P<0.001$.

seen in Figure 7, tumour growth was clearly slowed by treatment, more by liposome-delivered drug than by free drug. Groups treated with plain liposomes and immunoliposomes behaved similarly, plain liposomes being slightly more effective. Addition of unconjugated, soluble antibody to free DOX or plain liposomes, at doses equal to the amount of antibody given with immunoliposomes (~100 μg MAb per mouse), did not have any impact on the therapeutic effect (data not shown). Thus, antibody targeting of liposomes did not endow any therapeutic advantage over plain liposomes, nor was there a significant loss of activity.

peutic experiment, a significant number of toxic deaths were observed in the group of animals treated with DOX in non-targeted liposomes (12/18 mice died), in contrast to the other experimental groups (1/9 for DOX in immunoliposomes, 1/14 for free DOX, 0/6 for control untreated). All deaths occurred 3–5 weeks after the start of treatment and were preceded by severe weight loss. An additional group of six mice treated with DOX in liposomes lacking the PEG-hydrazide linker (Doxil) showed no signs of toxicity (0/6 deaths) and a level of anti-tumour activity at least as potent as that of the PEG-hydrazide-containing preparation (Table I). This is consistent with our past experience (Gabizon, 1992; Papahadjopoulos *et al.*, 1991), which indicates that the dosage of liposomal DOX used in this study is not toxic. These observations suggest that the PEG-hydrazide group in unquenched form (i.e. not protein bound) contributes to the toxicity observed, but not to the anti-tumour activity.

**Figure 7** Therapeutic efficacy of liposomal and free DOX on s.c. implanted N-87 tumours. Approximately $6\times10^6$ cells were injected into both flanks of nude mice. On days 15 (tumour volume of $300\pm150$ mg) and 22, i.v. treatment of 8 mg kg$^{-1}$ DOX was given as free or liposomal drug. Control is the non-treated group. Tumour volume was measured three times a week and estimated by the following equation $ab^2/2$, where $a$ is the largest tumour diameter and $b$ is the perpendicular diameter. ■, plain liposome DOX; ▲, immunoliposome DOX; ●, free DOX; □, control. Each group consisted of at least 12 tumours. Protein/phospholipid ratio, 52 μg μmol$^{-1}$. Addition of free antibody, at the same dose as the conjugated antibody (~100 μg per mouse) to DOX (free or liposomal), had no effect on tumour growth (data not shown).

### Discussion

Recent reports have indicated the feasibility of using immunoliposomes for targeted drug delivery to augment the therapeutic efficacy of an encapsulated anti-cancer drug (Ahmad *et al.*, 1993; Emanuel *et al.*, 1996). These models used DOX in Ab-targeted stealth liposomes, but their relevance to humans is limited as they are based on mouse tumours. We have tried to examine the likelihood that such a strategy could succeed in a human tumour model by targeting liposomes to erbB-2 oncoprotein, a receptor that is stably overexpressed by certain carcinomas and required for the maintenance of aggressive tumour growth (Berchuck *et al.*, 1990, Slamon *et al.*, 1987, 1989). Our aim was to develop stealth immunoliposomes equipped with anti-erbB-2 monoclonal antibodies, to preserve the pharmacokinetic properties of stealth liposomes and to improve tumour drug delivery and anti-tumour activity.

In this study, anti-erbB-2 MAb coupled to stealth liposomes using the Hz-PEG-DSPE linker was found to function as a specific and efficient targeting device in *in vitro* systems. This is in agreement with other studies using the same coupling system with a different antibody (Allen *et al.*, 1994; Zalipsky *et al.*, 1993*b*).

Experiments in biodistribution studies using nude mice bearing N-87 xenografts revealed that, despite optimal preparation of antibody-conjugated liposomes with high *in vitro* affinity to tumour cells and reduced RES uptake, no benefit of tumour liposome levels over plain stealth liposomes was noted. Therapeutic experiments correlated with biodistribution studies, i.e. similar anti-tumour efficacy of the two liposomal preparations was found. Despite the *in vitro* pronounced binding affinity of immunoliposomes to target cells, no improvement in therapeutic efficacy was achieved.

Baselga *et al.* (1996), in a phase II trial using humanised anti-erbB-2 MAbs, observed accelerated plasma clearance in patients with high plasma levels of extracellular domain of erbB-2 (ECD$^{HER2}$). Our studies detected minimal differences in plasma clearance rates between immunoliposomes and plain liposomes. Whether this is the outcome of low plasma levels of ECD$^{HER2}$ or of their minor effect on circulating α-erbB-2-targeted liposomes remains to be clarified.

Skin liposome uptake was extremely high for both liposome preparations, a finding consistent with the skin toxicity of a stealth liposomal DOX (Doxil) preparation in humans (Uziely *et al.*, 1995). High liposome localisation in skin has been reported previously in nude mice (Gabizon *et al.*, 1990; Huang *et al.*, 1993), although the underlying mechanism remains poorly understood.

These studies suggest that the rate-limiting factor of liposome accumulation in tumours is the liposome extravasation process, irrespective of liposome affinity or targeting to tumour cells. This hypothesis has the following rational basis. It is clear that the erbB-2-targeted antigens are to be found beyond the endothelial cell barrier. Liposomes need firstly to extravasate in tumour areas and, only then, does binding to tumour cell receptors occur. As the extravasation efficiency of plain liposomes should not be less than that of immunoliposomes, a difference in tumour accumulation can only be established if the former are washed out from the tumour area faster than the latter. However, given the relatively large size of liposomes and the lack of functional lymphatic drainage in tumours (Jain, 1989) it is likely that most extravasated vesicles will remain in the tumour site, whether cellbound or not, until they are degraded or cleared by scavenger cells. For complexes of smaller size than liposomes, for which return to the circulation is feasible, binding to an extravascular target cell will be an important determinant of tumour accumulation through decreased washout from the tumour. In fact, a number of soluble MAbs do show a selective enhancement of concentration in the targeted tumour in comparison with irrelevant MAb (Jakowatz *et al.*, 1985). In line with this, enhanced *in vivo* efficacy of N12A5 MAb against N-87 tumour (Hurwitz *et al.*, 1995), and of other anti-erbB-2 antibodies in various tumour models (Bacus *et al.*, 1992; Harwerth *et al.*, 1993), has been obtained.

A recent report (Park *et al.*, 1995) of a study with Fab′ fragments of anti-erbB-2 MAb (from a different source) conjugated via MPB-PE to the lipid bilayer of stealth liposomes demonstrates internalisation of immunoliposomes by human-derived breast carcinoma cells (SKBR3, erbB-2-positive) *in vitro* and augmented cytotoxicity of anti-erbB-2-targeted liposomes over plain liposomes. Unfortunately, the N-87 model did not show an increased *in vitro* sensitivity to immunoliposomes, suggesting lack of internalisation of the cell-bound immunoliposomes into the target cells, or liposomal drug localisation in a non-bioavailable cellular compartment. It should be noted that soluble anti-erbB-2 N12A5 MAb is internalised by N-87 cells *in vitro* to a great extent (85%) as shown by Hurwitz *et al.* (1995). These observations indicate differences in internalisation capacity among the various erbB-2-positive carcinoma cell-lines, or an advantage for Fab′ fragments over the bulkier whole IgG in facilitating internalisation. A third possibility is that the lower molar fraction of PEG in the liposomes used by Park *et al.* (1995) (2% PEG of total phospholipid) may have facilitated internalisation compared with the liposomes used here (7.5% PEG). However, even in the presence of 6.7% PEG, internalisation of liposomes is still possible, as demonstrated by Lee and Low, (1995), with folate-targeted stealth liposomes and cell lines overexpressing folic acid receptor. These authors also reported that internalisation was accompanied by an increase in the cytotoxicity of liposomal DOX. Whether as a result of the intrinsic properties of the target cells and their receptors, the density of the liposome PEG coating, or the nature and size of the ligand, internalisation may be a necessary step to enhance the cytotoxicity of DOX encapsulated in targeted liposomes, and hence translate targeting into enhanced efficacy. The argument for internalisation *in vivo* would be that drug released in the tumour intracellular compartment cannot escape from the tumour, while drug released in the interstitial fluid, as in the case of non-targeted liposomes, may still be partially washed out from the tumour and return to circulation.

A positive result of the therapeutic study was the enhanced efficacy of stealth liposomal DOX, with or without targeting antibodies, over free DOX. Similar observations have been made in a number of human xenograft models (Williams *et al.*, 1993; Vaage *et al.*, 1994). As emphasised in this study, the factors involved in the design of immunoliposomes are complex and unique to this system, and the results may not be extrapolatable to other tumour models, particularly in cases in which the drug becomes bioavailable in the intracellular compartment as a result of immunoliposome internalisation. Our results highlight the serious limitations of the antibody–liposome targeting approach to extravascular tumours. Nevertheless, the advantages of the liposome approach [multivalent binding and delivery of a large drug payload; the availability of long-circulating liposomes; an ever-increasing variety of ligand-coupling techniques; and the possibility of aiming at alternative targets such as the tumour microvasculature (Burrows and Thorpe, 1993) or intravascular targets] justify further investigation in this field.

### Abbreviations

DOX, doxorubicin; Chol, cholesterol; [$^3$H]Chol ether, [$^3$H]cholesteryl hexadecyl ether; mPEG, polyethylene glycol; Hz, hydrazide; DSPE, distearoyl phosphatidylethanolamine; MPB-PE, maleimidophenylbutyryl phosphatidylethanolamine; NAM, *N*-acetylmethionine, α-Toco, α-tocopherol.

### Acknowledgements

We are grateful to Dr Esther Hurwitz for helpful advice in the purification of antibodies, and to Dina Tzemach for technical help. Work supported by the Israel Ministry of Science and Technology and by Sequeus Pharmaceuticals (Menlo Pk., CA, USA).

### References

AHMAD I AND ALLEN TM. (1992). Antibody-mediated specific binding and cytotoxicity of liposome entrapped doxorubicin to lung cancer cells in vitro. *Cancer Res.*, **52**, 4817–4820.

AHMAD I, LONGENECKER M, SAMUEL J AND ALLEN TM. (1993). Antibody-targeted delivery of doxorubicin entrapped in sterically stabilized liposomes can eradicate lung cancer in mice. *Cancer Res.*, **53**, 1484–1488.

ALLEN TM, AGRAWAL AK, AHMAD I, HANSEN CB AND ZALIPSKY S. (1994). Antibody-mediated targeting of long circulating (Stealth) liposomes. *J. Liposome Res.*, **4**, 1–25.

BACUS SS, STANCOVSKI I, HUBERMAN E, CHIN D, HURWITZ E, MILLS GB, ULLRICH A, SELA M AND YARDEN Y. (1992). Tumor inhibitory monoclonal antibodies to the HER-2/neu receptor, induce differentiation of human breast cancer cells. *Cancer Res.*, **52**, 2580–2589.

BASELGA J, TRIPATHY D, MENDELSOHN J, BOUGHMAN S, BENZ CC, DANTIS L, SKLARIN NT, SEIDMAN AD, HUDIS CA, MOORE J, ROSEN PP, TWADDELL T, HENDERSON C AND NORTON L. (1996). Phase II study of weekly intravenous recombinant humanized anti-p185$^{HER2}$ monoclonal antibody in patients with HER2/neu-overexpressing metastatic breast cancer. *J. Clin. Oncol.*, **14**, 737–744.

BERCHUCK A, KAMEL A, WHITAKER R, KERNS B, OLT G, KINNEY R, SOPER JT, DODGE R, CLARKE-PEARSON DL AND MARKS P. (1990). Overexpression of HER-2/neu is associated with poor survival in advanced epithelial ovarian cancer. *Cancer Res.*, **50**, 4087–4091.

BURROWS FJ AND THORPE PE. (1993). Eradication of large solid tumors in mice with an immunotoxin directed against tumor vasculature. *Proc. Natl Acad. Sci. USA*, **90**, 8996–9000.

DEBS RJ, HEATH TD AND PAPAHADJOPOULOS D. (1987). Targeting of anti-thy 1.1 monoclonal antibody- conjugated liposomes in Thy 1.1 mice after intravenous administration. *Biochim. Biophys. Acta*, **901**, 183–190.

EMANUEL N, KEDAR E, BOLOTIN EM, SMORODINSKY NI AND BARENHOLZ Y. (1996). Targeted delivery of doxorubicin via sterically stabilized immunoliposomes: pharmacokinetics and biodistribution in tumor-bearing mice. *Pharm. Res.*, **13**, 861–868.

GABIZON A. (1992). Selective tumor localization and improved therapeutic index of anthracyclines encapsulated in long circulating liposomes. *Cancer Res.*, **52**, 891–896.

GABIZON A, PRICE DC, HUBERTY J, BRESALIER RS AND PAPAHADJOPOULOS D. (1990). Effect of liposome composition and other factors on the targeting of liposomes to experimental tumors: biodistribution and imaging studies. *Cancer Res.*, **50**, 6371–6378.

HARRIS DT AND MASTRANGELO MJ. (1989). Serotherapy of cancer. *Semin. Oncol.*, **16**, 180–198.

HARWERTH IM, WELS W, SCHLEGEL J, MUELLER M AND HYNES NE. (1993). Monoclonal antibodies directed to the erB-2 receptor inhibit *in vivo* tumour cell growth. *Br. J. Cancer*, **68**, 1140–1145

HOROWITZ AT, TREVES AJ, VOSS R, OKON E, FUKS Z, DAVIDSON L AND BIRAN S. (1985). A new human ovarian carcinoma cell line: establishment and analysis of tumor-associated markers. *Oncology*, **42**, 332–337.

HOROWITZ AT, BARENHOLZ Y AND GABIZON A. (1992). In vitro cytotoxicity of liposome-encapsulated doxorubicin: dependence on liposome composition and drug release. *Biochim. Biophys. Acta*, **1109**, 203–209.

HUANG SK, HONG K, LEE KD, PAPAHADJOPOULOS D AND FRIEND DS. (1991). Light microscopic localization of silver enhanced liposome entrapped colloidal gold in mouse tissues. *Biochim. Biophys. Acta*, **1069**, 117–121.

HUANG SK, MARTIN FJ, JAY G, VOGEL J, PAPAHADJOPOULOS D AND FRIEND DS. (1993). Extravasation and transcytosis of liposomes in Kaposi's sarcoma-like dermal lesions of transgenic mice bearing the HIV tat gene. *Am. J. Pathol.*, **143**, 10–14.

HURWITZ E, STANCOVSKI I, SELA M AND YARDEN Y. (1995). Suppression and promotion of tumor growth by monoclonal antibodies to ErbB-2 differentially correlate with cellular uptake. *Proc. Natl Acad. Sci. USA*, **92**, 3353–3357.

JAIN RK. (1989). Delivery of novel therapeutic agents in tumors: physiological barriers and strategies. *J. Natl Cancer Inst.*, **81**, 570–576.

JAKOWATZ GJ, BEATTY BG, VLAHOS WG, PORUDOMINSKY D, PHILBEN VJ, WILLIAMS LE, PAXTON RJ, SHIVLEY JE AND BEATTY JD. (1985). High-specific-activity $^{111}$In-labeled anticarcinoembryonic antigen monoclonal antibody: biodistribution and imaging in nude mice bearing human colon cancer xenografts. *Cancer Res.*, **45**, 5700–5706.

LEE RJ AND LOW PS. (1995). Folate-mediated tumor cell targeting of liposome-entrapped doxorubicin in vitro. *Biochim. Biophys. Acta*, **1233**, 134–144.

PAPAHADJOPOULOS D, ALLEN TM, GABIZON A, MAYHEW E, MATTAY K, HUANG SK, WOODLE MC, LASIC DD, REDEMANN C AND MARTIN FJ. (1991). Sterically stabilized liposomes: improvements in pharmacokinetics and anti-tumor therapeutic efficacy. *Proc. Natl Acad. Sci. USA*, **88**, 11460–11464.

PARK JG, FRUCHT H, LAROCCA RV, BLISS DPJ, KURITA Y, CHEN TR, HENSLEE JG, TREPEL JB, JENSEN RT, JOHNSON BE, BANG YJ, KIM JP AND GAZDAR AF. (1990). Characteristics of cell lines established from human gastric carcinoma. *Cancer Res.*, **50**, 2773–2780.

PARK JW, STAGG R, LEWIS GD, CARTER P, MANEVAL D, SLAMON DJ, JAFFE H AND SHEPARD HM. (1992). Advances in cellular and molecular biology of breast cancer. In *Genes, Oncogenes, Hormones*, Dickson RB and Lippman ME. (eds) pp. 193–211. Kluwer Academic Publishing: Boston.

PARK JW, HONG K, CARTER P, ASGARI H, GUO LY, KELLER GA, WIRTH C, SHALABY R, KOTTS C, WOOD WI, PAPAHADJOPOULOS D AND BENTZ CC. (1995). Development of anti-p185$^{HER2}$ immunoliposomes for cancer therapy. *Proc. Natl Acad. Sci. USA*, **92**, 1327–1331.

PEETERS PA, BRUNINK BG, ELING WM AND CROMMELIN DJ. (1989). Therapeutic effect of chloroquine (CQ)-containing immunoliposomes in rats infected with Plasmodium berghe: parasitized mouse red blood cells: comparison with combination of antibodies and CQ or liposomal CQ. *Biochim. Biophys. Acta*, **981**, 269–276.

PRESS MF, CORDON-CARDO C AND SLAMON DJ. (1990). Expression of the HER-2/neu proto-oncogene in normal human adults and fetal tissues. *Oncogene*, **5**, 953–962.

SLAMON DJ, CLARK GM, WONG SG, LEVIN WJ, ULLRICH A AND MCGUIRE WL. (1987). Human breast cancer: correlation of relapse and survival with amplification of the HER-2 neu oncogene. *Science*, **235**, 177–182.

SLAMON DJ, GODOLPHIN W, JONES LA, HOLT JA, WONG SG, KEITH DE, LEVIN WJ, STUART SG, UDOVE J, ULLRICH A AND PRESS MF. (1989). Studies of the HER-2/neu protooncogene in human breast and ovarian cancer. *Science*, **244**, 707–712.

STANCOVSKI I, HURWITZ E, LEITNER O, ULLRICH A, YARDEN Y AND SELA M. (1991). Mechanistic aspects of the opposing effects of monoclonal antibodies to the erbB-2 receptor on tumor growth. *Proc. Natl Acad. Sci. USA*, **88**, 8691–8695.

UZIELY B, JEFFERS S, ISACSON R, KUTSCH K, WEI-TSAO D, YEHOSHUA Z, MUGGIA FM AND GABIZON A. (1995). Liposomal doxorubicin: antitumor activity and unique toxicities during two complementary phase I studies. *J. Clin. Oncol.*, **13**, 1777–1785.

VAAGE J, BARBERA-GUILLEM E, ABRA R, HUANG A AND WORKING P. (1994). Tissue distribution and therapeutic effect of intravenous free or encapsulated liposomal Doxorubicin on human prostate carcinoma xenografts. *Cancer*, **73**, 1478–1484.

WILLIAMS SS, ALOSCO TR, MAYHEW E, LASIC DD, MARTIN FJ AND BANKERT RB. (1993). Arrest of human lung tumor xenograft growth in severe combined immunodeficient mice using Doxorubicin encapsulated in sterically stabilized liposomes. *Cancer Res.*, **53**, 3964–3967.

YONEMURA Y, NINOMIYA I, YAMAGUCHI A, FUSHIDA S, KIMURA H, OHOYAMA S, MIYAZAKI I, ENDOU Y, TANAKA M AND SASAKI T. (1991). Evaluation of immunoreactivity for erbB2 protein as a marker of poor short term prognosis in gastric cancer. *Cancer Res.*, **51**, 1034–1038.

ZALIPSKY S. (1993*a*). Synthesis of an end-group functionalized polyethylene glycol-lipid conjugate for preparation of polymer-grafted liposomes. *Bioconjug. Chem.*, *4*, 296–299.

ZALIPSKY S, NEWMAN M, PUNTAMBEKAR B AND WOODLE MC. (1993*b*). Model ligands linked to the polymer-chains on liposomal surfaces: application of a new functionalized polyethylene glycol–lipid conjugate. *Polym. Mater. Sci. Eng.*, **67**, 519–520.

ZALIPSKY S, PUNTAMBEKAR B, BOULIKAS P, ENGBERS CM AND WOODLE MC. (1995). Peptide attachment to extremities of liposomal surface grafted PEG chains: preparation of the long-circulating form of laminin pentapeptide, YIGSR. *Bioconjugate Chem.*, **6**, 705–708.